# The rapid identification of lactic acid bacteria present in Chilean winemaking processes using culture-independent analysis

**DOI:** 10.1007/s13213-014-0810-6

**Published:** 2014-01-25

**Authors:** Carolina Ilabaca, Carla Jara, Jaime Romero

**Affiliations:** 1 Laboratorio de Biotecnología, Instituto de Nutrición y Tecnología de los Alimentos (INTA), Universidad de Chile, El Líbano, 5524 Macul Santiago, RM Chile; 2 Departamento de Agroindustria y Enología, Facultad de Ciencias Agronómicas, Universidad de Chile, Av. Santa Rosa, 11315 La Pintana, Santiago, RM Chile

**Keywords:** Malolactic fermentation, *Oenococcus oeni*, Lactic acid bacteria, Wine, Chile

## Abstract

A polymerase chain reaction-restriction fragment length polymorphism (PCR-RFLP) analysis of 16S ribosomal RNA (rRNA) genes was developed to identify lactic acid bacteria (LAB) that are commonly present in winemaking processes (*Oenococcus, Pediococcus, Lactobacillus*, and *Leuconostoc*). This culture-independent approach revealed the presence of *Oenococcus* in the spontaneous malolactic fermentation in industrial Chilean wines.

**Electronic supplementary material:**

The online version of this article (doi:10.1007/s13213-014-0810-6) contains supplementary material, which is available to authorized users.

In Chile, the production of wines has sharply increased in recent years, reaching 12.5 million hL per year (Servicio Agrícola y Ganadero, Chile 2012). In most Chilean wineries, the malolactic fermentation (MLF) stage of winemaking largely occurs in a spontaneous manner; thus, at present, limited knowledge exists regarding the bacterial populations involved in this process. MLF is an important stage impacting the wine quality, in which lactic acid bacteria (LAB) transform malic acid into lactic acid and CO_2_, decreasing the overall acidity of a wine and proving microbiological stability. Hence, this study aimed to develop a rapid molecular method to describe LAB populations during spontaneous MLF.

A succession of LAB is observed during spontaneous MLF. In particular, LAB belonging to the genera *Lactobacillus, Leuconostoc, Oenococcus,* and *Pediococcus* have been found in wine (Lonvaud-Funel 1999; Reguant and Bordons 2003; Ruiz et al. 2010). *Lactobacillus, Pediococcus,* and *Leuconostoc* species gradually disappear as vinification proceeds. In contrast, *Oenococcus oeni* becomes increasingly apparent during the course of MLF (Spano et al. 2007). This dynamic of bacterial populations present during MLF has been studied using culture-dependent techniques (Reguant and Bordons 2003). These bacteria grow slowly in artificial media; as a result, the isolation of these bacteria requires long incubation times (>5 days), and the biochemical identification of these bacteria is tedious because it depends on the growth of the cultured strains. Therefore, these methods are unsuitable for the practical monitoring of bacteria during industrial vinification. Furthermore, it is challenging to utilize these methods to obtain an accurate picture of the dynamics of LAB during MLF because these bacteria generally exhibit low cultivability (Amann et al. 1995; Hugenholtz et al. 1998).

More recently, molecular methods based on analyzing DNA extracted from a sample (culture-independent methods) have been employed to circumvent this issue. Renouf et al. (2006) and Spano et al. (2007) applied denaturing gradient gel electrophoresis (DGGE) to the analysis of bacterial populations during MLF. This method is difficult to utilize routinely because the achievement of reproducible gradients using DGGE requires relatively sophisticated equipment and trained personnel. In this work, a culture-independent analytical approach is proposed that requires only simple equipment and can provide rapid results regarding the composition of bacterial populations present during industrial-level MLF. In this method, *Oenococcus, Leuconostoc, Pediococcus,* and *Lactobacillus* bacteria are identified using a polymerase chain reaction-restriction fragment length polymorphism (PCR-RFLP) approach that involves two restriction enzymes. This approach allows for the simultaneous determination of the presence or absence of the four most prevalent winemaking LAB genera in MLF during vinification.

The design of this MLF tracking method involved approximately 500 bp of the 16S ribosomal RNA (rRNA) gene sequences of the *Oenococcus, Leuconostoc, Pediococcus,* and *Lactobacillus* genera; in particular, numbering base pairs based on the *Escherichia coli*. 16S rRNA gene, the region between base pairs 341 and 788 was examined (Figure S1). These sequences were aligned with ClustalW (Larkin et al. 2007) and subjected to an entropy analysis to determine regions that differentiated the examined genera. Four such regions, located approximately at base pairs 456–461, 616–621, 651–656, and 766–771, were identified through this analysis; these regions are useful for using 16S rRNA gene analysis to distinguish among these four winemaking LAB genera.

The in silico design for the MLF tracking approach required the identification of restriction endonucleases that recognized sites within these regions. This search was performed using the BioEdit software program, version 7.1.3 (Hall 1999). Finally, two enzymes, *Xmn*I and *Tsp*RI, were selected. The simultaneous use of both enzymes produced distinctive restriction patterns that differentiate among the examined winemaking LAB. Comparing the in silico with the experimental restriction profiles of reference strains allowed the validation of the method to distinguish LAB populations. The reference strains from different collections were *Oenococcus oeni* JCM (Japan Collection of Microorganisms) 6125; *Leuconostoc mesenteroides* LMG (Laboratorium voor Microbiologie) 8159; *Pediococcus parvulus* NBRC (NITE Biological Research Center) 100673; and *Lactobacillus brevis* ATCC (American Type Culture Collection) 14687. To perform these comparisons, reference strains were grown in defined culture media, and DNA was extracted in accordance with Romero et al. (2002). The 16S rRNA gene was then amplified by PCR in accordance with Ilabaca et al. (2008). A combination of the restriction enzymes *Xmn*I (Thermo Scientific) and *Tsp*RI (Thermo Scientific), which were chosen based on the in silico analysis, was used for amplicon digestion. Amplicons were incubated with these restriction enzymes for 2 h at 37 °C and 16 h at 65 °C. The obtained restriction profiles were visualized using 90 min of electrophoresis at 80 V on 10 % polyacrylamide gels followed by staining with SYBR Green (Invitrogen).

The results obtained from these analyses are depicted in Fig. 1. The reference strains exhibit different profiles following digestion with the tested enzymes. These restriction enzymes, *Xmn*I and *Tsp*RI, provide results that are easy to visualize. In particular, a laddered pattern of restriction fragments is observed, with bands appearing at approximately 300 bp for *Leuconostoc*, 250 bp for *Oenococcus*, 200 bp for *Lactobacillus,* and 150 bp for *Pediococcus*. The exact sizes of the fragments are described in Table S1, it also describes that same genus always shows the same RFLP pattern.

**Fig. 1:**
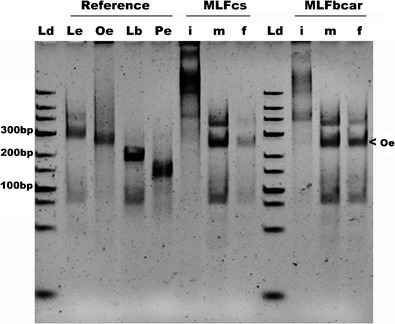
RFLP profiles derived from reference strains and illustrating the identification of LAB during the MLF stages of the production of Cabernet Sauvignon (cs) and Carménère (car). The reference strains (Reference) correspond to *Leuconostoc mesenteroides* LMG 8159 (Le), *Oenococcus oeni* JCM 6125 (Oe), *Lactobacillus brevis* ATCC 14687 (Lb), and *Pediococcus parvulus* NBRC 100673 (Pd). The analyzed samples correspond to the following MLF stages: the initial stage (i), the middle stage (m), and the final stage (f). Ld represents the O’GeneRuler Low Range DNA Ladder

The developed method was applied to wine samples during the MLF process. Spontaneous industrial MLF was monitored for *Vitis vinifera* cv. Cabernet Sauvignon and cv. Carménère (10,000 L). Different stages of the MLF process were examined: the initial stage of MLF (the iMLF lanes; this stage coincides with the end stage of alcoholic fermentation); the middle stage of MLF (the mMLF lanes), and the final stage of MLF (the fMLF lanes). For Cabernet Sauvignon, the initial stage of MLF was May 7, the middle stage of MLF was Jun 13, and the final stage of MLF was Jun 28. For Carménère, the initial stage of MLF was May 30, the middle stage of MLF was Jun 20, and the final stage of MLF was Jul 14. In the iMLF lanes (Fig. 1), the bands produced from samples of both cultivars do not correspond to bands produced by the LAB that are reportedly associated with MLF. This phenomenon may reflect the fact that only a limited LAB population is present during the initial stages of MLF; thus, this population may be below detection limits (Ruiz et al. 2010). In contrast, at the midpoint of MLF, the mMLF lanes for the examined cultivars exhibited bands indicative of the presence of *Oenococcus*. The presence of *Oenococcus* was also observed during the final stage of MLF (the fMLF lanes) for the examined cultivars. To confirm the identity of Oenococcus band, specific primers (F: GCTAAATACGTGCCAGCAGC; R: TCCACTTGCCTCTATCGCAC) were design and the band was eluted and sequenced; the resulting sequence corresponded to *Oenococcus* with 100 % of identity as derived from Blast analysis (see Figure S2). In summary, the profiles revealed the prevalence of *Oenococcus* during the MLF process, and bacteria from this genus were readily differentiable from the other genera involved in MLF.

Based on the aforementioned results, the designed 16S rRNA PCR-RFLP strategy constitutes a fast, easy, informative and reliable tool for the identification and differentiation of winemaking LAB strains isolated during MLF processes. The proposed approach can distinguish among LAB genera reported to be present during MLF (Lonvaud-Funel et al. 1991; Bon et al. 2009; Ruiz et al. 2010). Consequently, the 16S rRNA PCR-RFLP approach using *Xmn*I and *Tsp*RI allows for the simultaneous parallel observation of the presence or absence of the four genera (*Oenococcus, Leuconostoc, Pediococcus,* and *Lactobacillus*) of LAB that are most prevalent during the middle and final stage of MLF. This strategy can be used in both spontaneous MLF and MLF induced with commercial starter cultures. Thus, this qualitative method allows the genera of LAB involved in spontaneous MLF to be determined. In the case of MLF inoculated with commercial starter cultures, the introduction of the inoculated bacteria can be controlled, and any contamination by other winemaking LAB strains, which could alter the organoleptic characteristics of a wine, can be monitored.

## Electronic supplementary material

Below is the link to the electronic supplementary material.

Figure S1 (PDF 21 kb)

Figure S2 (PDF 18 kb)
