# Supplementary material for: The rapid identification of lactic acid bacteria present in Chilean winemaking processes using culture-independent analysis
Source: Ann Microbiol. 2014 Jan 25;64(4):1857–9. doi: 10.1007/s13213-014-0810-6 (PMC4234820; doi:10.1007/s13213-014-0810-6)
Supplement: Supplementary file 1 — (PDF 21 kb) [file 13213_2014_810_MOESM1_ESM.pdf]

Figure S1: Alignment of the compared 16SrRNA region of reference strains (ClustalW)

```
               ....|....| ....|....| ....|....| ....|....| ....|....|
                      10         20         30         40         50
Oenococus      CCTACGGGAG GCTGCAGTAG GGAATTTTCC GCAATGCACG AAAGTGTGAC
Pediococus     CCTACGGGAG GCAGCAGTAG GGAATCTTCC ACAATGGACG AAAGTCTGAT
Leuconosto     CCTACGGGAG GCTGCAGTAG GGAATCTTCC ACAATGGGCG AAAGCCTGAT
Lactobacil     CCTACGGGAG GCAGCAGTAG GGAATCTTCC ACAATGGACG AAAGTCTGAT

               ....|....| ....|....| ....|....| ....|....| ....|....|
                      60         70         80         90        100
Oenococus      GGAGCGACGC CGCGTGTGTG ATGAAGGCTT TCGGGTCGTA AAGCACTGTT
Pediococus     GGAGCAACGC CGCGTGAGTG ATGAAGGCTT TAGGGTCGTA AAACTCTGTT
Leuconosto     GGAGCAACGC CGCGTGTGTG ATGAAGGCTT TCGGGTCGTA AAGCACTGTT
Lactobacil     GGAGCAATGC CGCGTGAGTG AAGAAGGGTT TCGGCTCGTA AAACTCTGTT

               ....|....| ....|....| ....|....| ....|....| ....|....|
                     110        120        130        140        150
Oenococus      GTAAGGGAAG AATAACTGAA TTCAGAGAAA GTTTTCAGCT TGACGGTACC
Pediococus     GTTGGAGAAG AACGTGTGTG AGAGTAACTG CTCATGCAGT GACGGTATCC
Leuconosto     GTATGGGAAG AACAGCTAGA ATAGGAAATG ATTTTAGTTT GACGGTACCA
Lactobacil     GTTAAAGAAG AACACCTTTG AGAGTAACTG TTCAAGGGTT GACGGTATTT

               ....|....| ....|....| ....|....| ....|....| ....|....|
                     160        170        180        190        200
Oenococus      TTACCAGAAA GGGATGGCTA AATACGTGCC AGCAGCCGCG GTAATACGTA
Pediococus     AACCAGAAAG CCACGGCTAA CTACGTGCCA GCAGCCGCGG TAATACGTAG
Leuconosto     TACCAGAAAG GGACGGCTAA ATACGTGCCA GCAGCCGCGG TAATACGTAT
Lactobacil     AACCAGAAAG CCACGGCTAA CTACGTGCCA GCAGCCGCGG TAATACGTAG

               ....|....| ....|....| ....|....| ....|....| ....|....|
                     210        220        230        240        250
Oenococus      TGTCCCGAGC GTTATCCGGA TTTATTGGGC GTAAAGCGAG CGCAGACGGT
Pediococus     GTGGCAAGCG TTATCCGGAT TTATTGGGCG TAAAGCGAGC GCAGGCGGTC
Leuconosto     GTCCCGAGCG TTATCCGGAT TTATTGGGCG TAAAGCGAGC GCAGACGGTT
Lactobacil     GTGGCAAGCG TTGTCCGGAT TTATTGGGCG TAAAGCGAGC GCAGGCGGTT

               ....|....| ....|....| ....|....| ....|....| ....|....|
                     260        270        280        290        300
Oenococus      TTATTAAGTC TGATGTGAAA TCCCGAGGCC CAACCTCGGA ACTGCATTGG
Pediococus     TTTTAAGTCT AATGTGAAAG CCTTCGGCTT AACCGAAGAA GTGCATTGGA
Leuconosto     TATTAAGTCT GATGTGAAAG CCCGGAGCTC AACTCCGGAA TGGCATTGGA
Lactobacil     TTTTAAGTCT GATGTGAAAG CCTTCGGCTT AACCGGAGAA GTGCATCGGA

               ....|....| ....|....| ....|....| ....|....| ....|....|
                     310        320        330        340        350
Oenococus      AAACTGATTT ACTTGAGTGC GATAGAGGCA AGTGGAACTC CATGTGTAGC
Pediococus     AACTGGAAGA CTTGAGTGCA GAAGAGGACA GTGGAACTCC ATGTGTAGCG
Leuconosto     AACTGGTTAA CTTGAGTGCA GTAGAGGTAA GTGGAACTCC ATGTGTAGCG
Lactobacil     AACTGGGAGA CTTGAGTGCA GAAGAGGACA GTGGAACTCC ATGTGTAGCG

               ....|....| ....|....| ....|....| ....|....| ....|....|
                     360        370        380        390        400
Oenococus      GGTGAAATGC GTAGATATGT GGAAGAACAC CAGTGGCGAA AGCGGCTTGC
Pediococus     GTGAAATGCG TAGATATATG GAAGAACACC AGTGGCGAAG GCGGCTGTCT
Leuconosto     GTGGAATGCG TAGATATATG GAAGAACACC AGTGGCGAAG GCGGCTTACT
Lactobacil     GTGGAATGCG TAGATATATG GAAGAACACC AGTGGCGAAG GCGGCTGTCT

               ....|....| ....|....| ....|....| ....|....| ....|....|
                     410        420        430        440        450
Oenococus      TAGATCGTAA CTGACGTTGA GGCTCGAAAG TATGGGTAGC AAACGGGATT
Pediococus     GGTCTGTAAC TGACGCTGAG GCTCGAAAGC ATGGGTAGCG AACAGGATTA
Leuconosto     GGACTGCAAC TGACGTTGAG GCTCGAAAGT GTGGGTAGCA AACAGGATTA
```

```
Lactobacil   AGTCTGTAAC TGACGCTGAG GCTCGAAAGC ATGGGTAGCG AACAGGATTA


             ....|....| ....|..
                   460
Oenococus    AGATACCCCG GTAGTCC
Pediococus   GATACCCTGG TAGTCC.
Leuconosto   GATACCCTGG TAGTCC.
Lactobacil   GATACCCTGG TAGTCC.
```
