# Supplementary material for: The rapid identification of lactic acid bacteria present in Chilean winemaking processes using culture-independent analysis
Source: Ann Microbiol. 2014 Jan 25;64(4):1857–9. doi: 10.1007/s13213-014-0810-6 (PMC4234820; doi:10.1007/s13213-014-0810-6)
Supplement: Supplementary file 2 — (PDF 18 kb) [file 13213_2014_810_MOESM2_ESM.pdf]

Figure S2.- Alignment of the sequence of Oe bands reamplified using specific primers

```
               ....|....| ....|....| ....|....| ....|....| ....|....|
                        10         20         30         40         50
Banda Oeni     CGAGCGTTAT CCGGATTTAT TGGGCGTAAA GCGAGCGCAG ACGGTTTATT
Banda CS m     CGAGCGTTAT CCGGATTTAT TGGGCGTAAA GCGAGCGCAG ACGGTTTATT
banda CS f     CGAGCGTTAT CCGGATTTAT TGGGCGTAAA GCGAGCGCAG ACGGTTTATT
Banda CR m     CGAGCGTTAT CCGGATTTAT TGGGCGTAAA GCGAGCGCAG ACGGTTTATT
Banda CR f     CGAGCGTTAT CCGGATTTAT TGGGCGTAAA GCGAGCGCAG ACGGTTTATT

               ....|....| ....|....| ....|....| ....|....| ....|....|
                        60         70         80         90        100
Banda Oeni     AAGTCTGATG TGAAATCCCG AGGCCCAACC TCGGAACTGC ATTGGAAACT
Banda CS m     AAGTCTGATG TGAAATCCCG AGGCCCAACC TCGGAACTGC ATTGGAAACT
banda CS f     AAGTCTGATG TGAAATCCCG AGGCCCAACC TCGGAACTGC ATTGGAAACT
Banda CR m     AAGTCTGATG TGAAATCCCG AGGCCCAACC TCGGAACTGC ATTGGAAACT
Banda CR f     AAGTCTGATG TGAAATCCCG AGGCCCAACC TCGGAACTGC ATTGGAAACT

               ....|....| ....|....| ....|....| .
                       110        120        130
Banda Oeni     GATTTACTTG AGTGCGATAG AGGCAAGTGG A
Banda CS m     GATTTACTTG AGTGCGATAG AGGCAAGTGG A
banda CS f     GATTTACTTG AGTGCGATAG AGGCAAGTGG A
Banda CR m     GATTTACTTG AGTGCGATAG AGGCAAGTGG A
Banda CR f     GATTTACTTG AGTGCGATAG AGGCAAGTGG A
```
